# Violence and Cannabis Use: A Focused Review of a Forgotten Aspect in the Era of Liberalizing Cannabis

**DOI:** 10.3389/fpsyt.2020.567887

**Published:** 2020-09-16

**Authors:** Laura Dellazizzo, Stéphane Potvin, Maria Athanassiou, Alexandre Dumais

**Affiliations:** ^1^Research Center of the Institut Universitaire en Santé Mentale de Montréal, Montreal, QC, Canada; ^2^Department of Psychiatry and Addictology, Faculty of Medicine, Université de Montréal, Montreal, QC, Canada; ^3^Institut national de psychiatrie légale Philippe-Pinel, Montreal, QC, Canada

**Keywords:** cannabis use, violence, meta-analyses, legalization, public health

## Abstract

There has been a shift surrounding societal and legal perspectives on cannabis reflecting changing public attitudes towards the perceived safety and social acceptability of cannabis use. With cannabis liberalization internationally, the focus of most cannabis-related harms has been on effects with users themselves. Harm-to-others including injuries from violence have nevertheless been unfortunately largely overlooked. While studies remain heterogeneous, there is meta-analytical evidence pointing towards an association. The aims of this focused review are two-fold: (I) review the evidence from meta-analyses on the association between cannabis and violence; and (II) provide an overview of possible mechanisms relating cannabis use to violence. First, evidence from meta-analytical studies in youths, intimate partners, and individuals with severe mental disorders have shown that there is a global moderate association between cannabis use and violence, which is stronger in the latter more at-risk population. Preliminary data has even highlighted a potential dose-response relationship with larger effects in more frequent users. Although of importance, this subject has remained essentially forgotten as a public health concern. While literature remains inconclusive, data has suggested potential increases in cannabis use following liberalization policies. This may increase violent outcomes if the effect is directly related to the use of cannabis by means of its psychophysiological modifications. However, for the moment, the mechanisms associating cannabis use and violence remain to be clearly resolved. Considering the recency of policy changes on cannabis, further methodologically sound research using longitudinal designs should examine the effects that cannabis use may have on different forms of violence and the trends that emerge, while evaluating the effects of possible confounding factors (e.g. other substance use). In addition, as evidence-based research from meta-analyses have shown that cannabis use is associated with violence, measures must be taken to mitigate the risks.

## Introduction

Worldwide populational data shows that roughly 200 million individuals have used cannabis in the past year (1) and 13 million have a cannabis use disorder (CUD) (2). In recent years, there has been a shift surrounding societal and legal perspectives on cannabis reflecting changing public attitudes towards the perceived safety and social acceptability of its use (3). There is thus a growing number of U.S. states (e.g. Washington, Colorado) and countries (e.g. Portugal, Canada, Netherlands) that have liberalized their cannabis laws by decriminalizing (i.e. lessening the penalties for cannabis offenses) or legalizing its use for medical or recreational purposes (3, 4). Following these policy changes, although literature remains inconclusive and very preliminary with some studies having found no effect, there is some evidence that has also suggested a certain increase of cannabis use in some age groups such as young adults and older adult populations (4–7). Some data likewise suggested changes in frequency of use following recreational cannabis legalization in the U.S. with findings showing a small increase in adolescent CUD and increases in past-month cannabis use, past-month frequent cannabis use, and past-year CUD among adults over 26 years (8). Of note, studies, furthermore, suggest that cannabis has grown more potent as measured by the proportion of Δ^9^-tetrahydrocannabinol (THC) content in relation to cannabidiol (CBD) content (THC to CBD ratio) (9, 10). Accordingly, with policy changes, there has been increased attention into cannabis-related harms such as motor vehicle accidents, emergency medical attendances and hospitalizations, severe mental disorders (SMD) as well as suicides (1, 7). Harm-to-others including injuries from violence have nevertheless been unfortunately largely overlooked (11).

Violence is a complex and multifactorial issue that has serious health and social consequences (12). The association between cannabis and violence has created a range of debates. Although studies remain heterogeneous [i.e. (13–20)], there is meta-analytical evidence pointing towards an association. Particularly with liberalization policies aiming for public health and safety while using cannabis, harm-to-others should constitute an essential element for outcome monitoring (7, 11). The aims of this focused review are two-fold: (I) review evidence from meta-analyses on the association between cannabis and violence; and (II) provide an overview of possible mechanisms relating cannabis use to violence.

## Reviewing Evidence on the Cannabis-Violence Association

### Meta-Analytical Evidence

**Figure 1:**
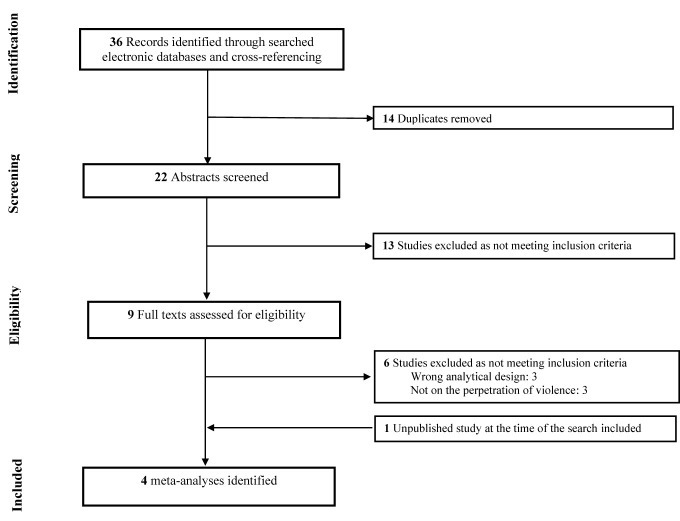
Flow-chart depicting the search strategy employed to find the meta-analyses included in this review.

Our team conducted a systematic search of literature in the online databases of PubMed, PsycINFO, Web of Science and Google Scholar to identify all relevant research reporting on the cannabis-violence relationship with no restriction as to the type of population being investigated. Additional records were identified through cross-referencing. Searches used key words that were inclusive for violence [e.g. (aggression, violent)] and cannabis use [e.g. (marijuana, cannabis)]. The search syntax was tailored for each database. No setting, date or geographical restrictions were applied. Searches were limited to English and French language sources and meta-analytical study designs. The Preferred Reporting Items for Systematic Reviews and Meta-Analyses (PRISMA) flowchart for the inclusion of meta-analyses within this review is found in **Figure 1**.

Below is a description of findings from meta-analyses in (i) youths and emerging adults, (ii) intimate partners, and (iii) individuals with SMD. To ensure clarity, the following qualitative descriptions of the strength of reported effects were used for (i) Odds Ratio [OR (21); small = 1.0–1.5, moderate = 1.6–2.5, strong = 2.6–9.9, and very strong = ≥ 10.0] and (ii) Cohen’s d [d (22); small = 0.2, medium = 0.5, and large = > 0.8].

#### Youths and Emerging Adults

Our team chose to conduct a meta-analysis to clarify the association between cannabis use and violence, more precisely, the perpetration of any type of physical violence by adolescents and young adults (23). Studies were included so long as the behaviors being reported comprised acts of physical violence (e.g. aggravated assault, sexual aggression, fighting, robbery). Studies were excluded if the definition of violence was unclear or included other types of behaviors (e.g. delinquency, verbal aggression, victimization, suicidality). As for cannabis use, all types of frequency measures (e.g. lifetime, occasional, frequent use) were extracted to examine a potential “*dose-response*” relationship in our sub-analyses. Based on this meta-analysis of 30 study arms, a moderate association between cannabis use and the perpetration of physical violence was observed [OR = 2.11, Confidence interval (CI) = 1.64–2.72]. This emerged from studies amounting from a large sample of 296,815 adolescents and young adults and showing no publication bias. It is, however, important to note that there was a high level of heterogeneity between studies, which may be due to the heterogeneous methods used in studies to measure and define physical violence. A challenge in the interpretation of findings is to rule out alternative explanations on the association itself and its direction, which this meta-analysis has attempted to do with the sub-analyses. First, preliminary findings on the effects of frequency do suggest a potential dose-response relationship, while mostly driven by two studies reporting high ORs (24, 25). More specifically, frequent, persistent and long-term users (i.e. early onset cannabis users) have been shown to experience more mental health and behavioral problems, such as aggression and delinquency (25–28). Beyond frequency of use, current studies did not conduct a detailed assessment of cannabis exposure/usage patterns (e.g. type of cannabis, number of joints, dosage, cannabis potency) (29), which may differentially be associated with violence. Second, the effect remained significant when considering studies additionally adjusting for several covariates including sociodemographic variables and other important confounding factors that may have better explained the relationship (e.g. other substance use such as alcohol, stimulants, conduct problems or psychopathic traits and prior violence) (30). Importantly, results showed that the effect size estimates did not differ substantially between studies that controlled for confounders versus those that did not (OR = 2.01 and OR = 2.62, respectively), meaning that the association is unlikely to be fully explained by confounders. Third, concerning the directionality of the association, we performed a sub-analysis with available data specifically from longitudinal studies and findings showed that cannabis use during adolescence may indeed lead individuals to perpetrate physical violence in early adulthood (OR = 2.02). Of note, the results from longitudinal studies may also be attributed to reverse causality (31, 32). A limited number of authors have indeed reported findings consistent with reverse causality suggesting that physical violence in adolescents and young adults may increase the risk of initiating the use of cannabis later in life (27, 31–33). This still needs further investigation.

#### Intimate Partners

Physical dating violence perpetration is an example of a behavioral problem that could be influenced by cannabis use in youths as well as in adults. A meta-analysis by Johnson et al. (34) focused on U.S. adolescents and emerging adults aged 11 to 21 and defined physical dating violence as any non-sexual physically aggressive behavior among current or former romantic, sexual/intimate or dating partners. They retrieved 11 studies with six on adolescents and five on emerging adults, which provided evidence for an association between cannabis use and violence perpetration. Globally, there was a 45% increase in the odds of perpetration (OR = 1.45, CI = 1.20–1.76) in cannabis users. As observed in the meta-analysis above, there was minimal evidence of publication bias, but a substantial amount of heterogeneity between studies. As stated by the authors of the meta-analysis, this was mostly the case of five included studies with methodological differences focusing on emerging adults. In comparison to adolescent literature, these latter studies comprised heterogeneous samples (e.g. 60% on college students, at least 70% Caucasians), a variety of study designs (e.g. cross-sectional, longitudinal, daily diary) and most adjusted for alcohol use. Another review by Moore et al. (35) quantitatively evaluated the empirical evidence on the relationship between several types of drugs, including cannabis, and partner aggression perpetration (psychological aggression, physical abuse, sexual coercion/abuse, and mixed forms) in a variety of populations (e.g. substance abuse treatment facilities, community samples). In the 15 studies retrieved for cannabis use, a small effect size (d = 0.22, CI = 0.21–0.28) was found for all types of interpersonal violence including psychological, physical, sexual abuse, and mixed. Effect sizes were larger for psychological aggression broadly defined (d = 0.35, CI = 0.19–0.50), and physical aggression (d = 0.21, CI = 0.14–0.27) in comparison to other forms of aggression. Notably, men’s use of cannabis was positively related to the perpetration of aggression. This study found that the relationship between cannabis use and intimate partner aggression was stable and reflected little variability in the effect sizes across studies. While both these meta-analyses found a positive association between cannabis use and violence, unfortunately, with the limited studies included, they did not conduct supplementary sub-analyses to further examine the direction of the association.

#### Individuals With Severe Mental Disorders

We conducted a meta-analysis to examine the association between cannabis use/misuse and the perpetration of violence in adult individuals with SMD (schizophrenia, schizophreniform disorder, schizoaffective disorder, delusional disorder, bipolar disorder, and major depression) (36). Notably, these individuals are already at an elevated risk of violence in comparison to the general population (37, 38). To be as inclusive as possible, studies were not restricted so long as they evaluated any type of violence/aggression by any means such as clinical observation and self-reports. The meta-analysis included 12 final articles amounting to a total of 3,873 subjects. Results showed a moderate association between cannabis use and violence in individuals with SMD (OR = 3.02, CI = 2.01–4.54). As observed in the other meta-analyses, there was no publication bias, however, the database was characterized by high heterogeneity. This may partly be due to the studies displaying a variety of definitions for violence and assessment methods. Importantly, to determine whether other factors may have modified the effect, we also conducted sub-analyses. When considering adjusted studies only, the effect was slightly smaller, but remained significant (OR = 2.82, CI = 1.89–4.23). The four studies adjusted for several factors including sociodemographic variables and other confounding factors such as substance use and presence of psychiatric disorders. Of clinical interest, the association was significantly higher for cannabis misuse in comparison to cannabis use (OR = 5.8, CI = 3.27–10.28 versus OR = 2.04, CI = 1.36–3.05). In contrast to our meta-analysis in youths, this frequency association was not driven by any individual studies. Beyond frequency of use, it was not possible to examine other cannabis exposure patterns (e.g. type of cannabis, dosage, potency). Moreover, since most data was cross-sectional and retrospective, evidence was limited as a basis for concluding on the direction of the association. Longitudinal studies examining the association between cannabis use and violent behavior in patients with SMD are critically needed.

**Table:** 

**Summary: Public health significance of evidence** **• **There is a *moderate* association between cannabis use and physical violence in youths and emerging adults, with a potential *dose-response* association. Moreover, longitudinal evidence suggests that cannabis use may lead to future violent outbursts. **• **There is a *small to moderate* association between cannabis use/misuse and intimate partner aggression perpetration. **• **There is a *moderate* association between cannabis use and violence in populations with severe mental disorders, with a significant increase for frequent users or those with a cannabis use disorder. **• **Evidence highlights that violence should be an *important indicator to monitor* considering recent cannabis liberalizations in several countries.

## Overview of Potential Mechanisms Explaining Violent Behavior and the Potential Impact With Cannabis Legalization

Harm-to-others such as violence constitutes an essential outcome to monitor in a public health perspective (7, 11). There are two main positions that have prevailed as to the consequence cannabis use policies might have on violence outcomes that depends chiefly on the impact these policies have on cannabis use as well as the mechanism by which cannabis and violence are associated (e.g. psychophysiological effects versus social context described below). Hence, although literature remains inconclusive, it has been hypothesized that there may be an increase in the number of cannabis users following the legalization of medical and recreational cannabis more particularly for adult samples (4–7, 39). Accordingly, for illustrative purposes, considering an expected increase of cannabis use:

- A rise in the rate of violence may be observed if the mechanisms involved is psychophysiological (e.g. increase of aggression-related effects while intoxicated or during withdrawal) Or
- A reduction in the risk of violence may be observed if the mechanisms involved is social (e.g. reduction of black-market-, gang-related violence).

The following describes both these mechanisms and briefly explores the support for these mechanisms from literature on the legalization of recreational cannabis in the U.S. Markedly, the first four states to legalize cannabis for recreational use were Colorado and Washington in 2014 and Alaska and Oregon in 2015.

### Psychophysiological Mechanisms

From a neurobiological perspective, cannabinoid receptors, CB-1 and CB-2, bind endogenous ligands, primarily anandamide and 2-arachidonoylglycerol to modulate neural activity (40). Amid receptors, CB-1 receptors are the predominant cannabinoid receptor type within the central nervous system and have been shown to mediate the effects of exogenous cannabinoids (41, 42). The main active ingredient in cannabis, THC, acts as a partial agonist for CB-1 receptors in the brain (43). With a lower efficacy than at CB-1 receptors, THC also demonstrates partial agonist properties for CB-2 receptors (44). CB-1 receptors are abundant in several cerebral regions, such as the cerebellum, basal ganglia, cingulate cortex, amygdala, hippocampus and frontal cortex that participate in several functions (e.g. executive, emotional, reward, and memory processing) (40, 45). Such brain function modulation occurs *via* direct interactions with the endocannabinoid system and indirect effects on neurotransmitter systems including the glutamatergic, GABAergic and dopaminergic systems (40, 45). Animal studies have shown that THC produces morphological changes (e.g. reductions in synapses, cell body size and dendritic length) in these brain regions with high CB-1 receptor expression (46–50).

Animal studies have found that THC produces complex effects on aggression. Indeed, animal studies have not produced clear-cut results, as both anti-aggressive as well as aggressive-inducing effects of THC have been documented [see (51–53) for reviews]. Discrepant results are likely related to several laboratory factors with the dose, delivery of administration and concurrent environmental manipulations being prominent aspects to consider. Based on a review of animal studies (52), it has been generally found that studies using smaller doses of THC/cannabis have been less likely to report the emergence of aggression, whereas studies using higher doses and more chronic exposure have rather led to an increase in aggressiveness. Such dose-dependent effects on aggression have been stated to be due to the fact that CB-1 agonists at low doses may increase serotonin (a key neurotransmitter system derived mainly from dorsal and medial raphe involved in aggression control), while at higher doses, they may induce a decrease of serotonin, thereby increasing aggression (54). In addition, experiments with genetically modified animal models, such as mice, lacking CB-1 receptors (CB-1KO) have also revealed alterations in the regulation of emotion and aggressive behaviors (55). For instance, CB-1KO mice exhibited stronger aggressive responses than wild-type mice when exposed to social interaction tests (56, 57). This may be explained by differences in serotonin that were observed in CB-1KO mice. While they appeared to better metabolize serotonin due to an increase in catechol-O-methyltransferase levels in the raphe nucleus and amygdala, gene expression of monoamine oxidase-A was also augmented in the amygdala, which may have reduced serotonin levels leading to increased aggressiveness (57). This supports the role of CB-1 receptors in aggressive behaviors. In all, animal models are necessary since they allow to generate hypotheses and may provide some parallels to aggression in humans (53). Although such findings on animal studies in controlled laboratory environments do not necessarily translate to human studies, they provide evidence of a relationship between CB-1 receptor and aggressive states.

Similar to animal models, alterations in brain regions have been observed in human studies, particularly in CB-1 receptor rich areas mediating not only executive and cognitive functions, but also emotional and affective processing [see (58) for a review]. These alterations in humans may lead to aggressive tendencies. While functional imaging studies on aggression as an outcome per se in association to cannabis use are lacking in human literature, changes observed in key regions involved in emotional processing such as the amygdala and the anterior cingulate cortex may be relevant to the regulation of negative emotions such as anger and hostility. Several studies have indeed found that acute cannabis use may alter the activity of these regions when presented with stimuli of negative valence, notably threatening stimuli (e.g. fearful and angry valence) (59–65). For instance, it was found that inhaling 6 mg of THC impaired task performance for matching emotional faces with negative emotional content, but not those with positive content (59). While processing stimuli with a negative emotional content, there was a reduction in neural activity in a network of brain regions including the amygdala, orbitofrontal gyrus, hippocampus, and prefrontal cortex. A further study showed that THC reduced the functional coupling between the basolateral amygdala with the rostral anterior cingulate cortex and the superficial amygdala with the medial prefrontal cortex (62). It is worth noting that the net effects of orally administered THC and CBD on amygdala activation during the processing of fearful faces have shown to be in the opposite direction (64). Further evidence of emotion dysregulation after chronic cannabis use is provided in functional imaging studies (66–70). Reductions in response within the cingulate, frontal cortex, and the amygdala during the presentation of negative emotional stimuli have been observed in literature on chronic cannabis use (68, 70). While passively exposed to negative and neutral valence pictures, negative emotional stimuli produced hypoconnectivity between the amygdala and dorsolateral prefrontal cortex in active users and orbitofronto-striatal and amygdala hyper-connectivity following 28 days of abstinence (67). Overall, cannabis users appear to process emotional stimuli differently in comparison to non-users and this may explain their impairment in the recognition of affect (68). Therefore, neutral stimuli can attain emotional/affective salience during the use of cannabis (71). Deficits in emotion recognition have been associated with violence (72, 73) and thus cannabis use inducing such impairments may increase the risk of violent acts. At the moment, the potential association between cannabis-induced changes in neural functioning and violent behavior in humans remains speculative, and future fMRI studies will need to directly measure levels of irritability and/or aggressiveness in cannabis users to determine if there is an association or not.

Compared to the general adult population, youths are particularly vulnerable to the neural effects of cannabis that is worthy of discussion. Preclinical studies have evidenced that the endocannabinoid system matures slowly during development, with maximal CB-1 receptor abundance achieved during adolescence, and that this system plays a key role in neural refinement during adolescence (74). More precisely, it has been shown that the chronic activation of CB-1 receptors by exogenous cannabinoids during adolescence could disrupt the maturation of GABAergic interneurons in the prefrontal cortex and disrupts the GABA-glutamate balance (75, 76). As a result, youths may be more vulnerable to the adverse consequences of cannabis use. In human literature, reviews have concluded that frequent cannabis use in adolescents and young adults is associated with anomalies in brain structure, including alterations in the basal ganglia, hippocampus, amygdala, cerebellum, cingulate cortex, and prefrontal cortex (58, 77–79). The findings suggest that earlier initiation of cannabis use is associated with more prominent alterations (79). Thus far, the most consistent alterations produced by cannabis use, mostly its chronic use, during youth have been observed in the prefrontal cortex. Such alterations may potentially lead to a long-term disruption of cognitive and executive functions (80). Interestingly, early and frequent cannabis use in adolescence predicts poor cognition and even emotional processing in adulthood (81), which may increase the likelihood of aggressiveness later in life. There are indeed indications that continued exposure to cannabis in youths is associated with a higher risk of subsequent violent behavior in later adulthood (27).

At the behavioral level, both acute and chronic cannabis intoxication may (i) impair neurocognitive domains (e.g. executive functioning) and create perceptional distortions (e.g. interpreting neutral actions as aggressive), (ii) impair a user’s ability to suppress aggressiveness, (iii) heighten physiological arousal making users feel paranoid, anxious or panicky (35). Withdrawal symptoms, which are reported by up to a third of regular users are of clinical significance as they can be impairing and associated with trouble ceasing use (82). These symptoms typically onset within 24 to 48 h following abrupt cessation in frequent users and contribute to irritability, restlessness, and anxiety that may likewise be associated with aggression (35, 83). These effects apply to psychiatric samples such as those with SMD as well. Both the acute intoxication and chronic use, in addition to the effects stated above, may lead to poor clinical outcomes and interfere with treatment by worsening and promoting psychiatric symptoms (84–86). Early regular and frequent cannabis use has been shown to be associated with onset of psychosis and worsens the course of the disorders (87, 88). Moreover, cannabis use may exacerbate psychotic symptoms such as delusions, which, in combination with the intoxicating effects of cannabis, may increase the risk of violence (13, 35). It is essential to note that individuals with SMD are also more likely to use cannabis and have comorbid substance use disorders in comparison to the general population (5, 89–93). This may reflect an attempt to cope with psychological distress (e.g. negative affective symptoms) or relieve the side effects of medication (e.g. antipsychotics) through cannabis use (e.g. self-medication) (94). Given the risks of continued substance use, it is important to identify the emergence of problematic use even more so as this population is at an increased risk of exhibiting aggressive behavior (37, 38). Lastly, distal influences (e.g. psychiatric disorders, childhood abuse, history of substance use) in concurrence with proximal factors (e.g. acute intoxication, impulsivity, emotional reactivity, encounter setting) may help to explain the increase in the risk for aggression when in the context of a conflictual interaction (35, 95). For example, cannabis intoxication in individuals with stable personality traits such as hostility and callousness may lead them to act aggressively when triggered in a fight. Although, it is worth noting that it is not only the psychophysiological effects of cannabis use per se that might induce violence, but also factors associated with substance use in general. As an example, the use of substances and related environments may lead to relational frictions, thereby increasing the chances of violence in conflictual circumstances (35).

#### Support From Cannabis Legalization Literature

A few scholars have recently found results showing that legalizing recreational cannabis may increase violence. Hughes et al. (96) assessed the relationship between both medical as well as recreational cannabis dispensaries and yearly neighborhood crime in Denver between 2012 and 2015, including the two-year period immediately following commencement of legal retail sales in January 2014. This was examined by controlling for correlates of neighborhood crime, including socioeconomic disadvantage and the concentration of high-risk commercial establishments. The authors found that the presence of at least one medical/recreational cannabis dispensary was associated with a statistically significant increase in neighborhood crime (e.g. robbery and aggravated assault). At the state-level, Lu et al. (97), comparing rates of crime in Washington and Colorado to states not legalizing cannabis, found some immediate increases in crime at the point of recreational legalization. Moreover, Lin et al. (98) conducted a non-peer reviewed quasi-experimental difference-in-difference analysis to study the potential effect of cannabis use on domestic violence by exploiting municipal and temporal variations in the enactment of recreational cannabis laws in Denver-Aurora-Lakewood Metropolitan Statistical Area from 2011 to 2016. They found that the enactment of recreational cannabis laws in 2014 led to a substantial increase in domestic violence. Denver and Aurora experienced a 48.2% increase in domestic violence rate as compared to their two control cities. Since the legal age to procure recreational marijuana is 21 years old, they even observed that the effect was only significant for perpetrators over that age. The effect was significant across gender and ethnic groups. As for offence severity, the effect concentrated for categories of simple assault, intimidation, minor injury, and no injury. As alcohol interacts with cannabis use, the authors found that the main findings were not driven by co-use of alcohol and cannabis.

### Social Mechanism

Supplementary explanations relate to the interaction between people and their social environments specifically. In jurisdictions where cannabis is illegal, users may obtain cannabis in the black market, thereby potentially exposing individuals to the risk of violence (99). The association between cannabis use and violence perpetration could be more broadly situational. For instance, selling or purchasing cannabis may promote criminal behavior for economic motives or to sustain substance use behaviors. While this may seem less relevant for intimate partners, relationships could be placed at risk of intimate partner aggression by supporting a habit related to use (e.g. stealing money) or by means of procuring a substance (e.g. forcing a partner to obtain a substance) (95). Aggressive tendencies may also occur within the broader system of drug use within the black-market (e.g. disputes over neglecting to pay debts) (95, 100). Legalizing recreational cannabis would ensure that citizens can procure the substance in places not governed by organized crime. Consequently, consumers would be less likely exposed to violent/criminal lifestyles.

#### Support From Cannabis Legalization Literature

Further analyses of recreational law reforms may best demonstrate whether eliminating the cannabis black-market might affect violent and property crime. Research has therefore also found support for the claim that legalizing recreational cannabis may reduce violent outcomes. Brinkman et al. (101) observed reductions on crime rates in geographical proximity to cannabis dispensaries in Colorado. There were no significant effects in crime on neighboring dispensary density. They found that a supplementary dispensary in a neighborhood led to a decline of 17 crimes per month per 10,000 citizens. This finding corresponded to a nearly 19% reduction in relation to the typical crime rate. The effect was generally stronger for nonviolent crimes (e.g. criminal trespassing, public-order crimes, criminal mischief, and simple assault). Dragone et al. (102) further examined crime rates from 2010 to 2014 in counties along the Washington-Oregon border before and after legalization in Washington. They used a quasi-experiment research design that combined a difference-in-difference design (where Washington acted as the treatment group, Oregon as the control group, 2010–2012 was the pre-legalization period and 2013–2014 was the post-legalization period) and spatial regression discontinuity designs (where the border marked a discontinuity in the legal status of cannabis in 2013–2014). The authors noted significant drops in rape and property crime in Washington side counties relative to Oregon-side counties. The study by Lin et al. (98) did find reductions in high gang-related crimes including aggravated assault and robbery, supporting the social mechanism as well. Moreover, Lu et al. (97) used a quasi-experimental, multi-group interrupted time-series design to examine crime rates in Colorado and Washington and determine if and how these rates were influenced by the legalization of recreational cannabis in 2012 and the beginning of retail sales in 2014. This study suggested that cannabis laws more broadly, and the legalization of recreational cannabis, have had minimal effects on major crime. While there were some short-term increases as stated in the section above, these did not result in long-term effects. They observed no statistically significant long-term effects apart from a significant decrease of burglary in Washington.

### Summary of Findings

Overall, there is evidence demonstrating an increase as well as a decline in general criminality/violence following the legalization of recreational cannabis, thus supporting both mechanisms. Under the first paradigm, research reinforces that legalizing cannabis policies may be expected to show a potential increase in cannabis use (while literature remains inconclusive in this regard) and may alter some users’ behavior, thereby increasing aggression. Under the second paradigm, the underground cannabis market intertwined with criminality is expected to diminish as the cannabis market becomes legalized. It may be possible that both a rise and reduction in different violent outcomes may emerge following cannabis legalization since both the psychophysiological and social effects can occur simultaneously as has been observed in the study by Lin et al. (98). The limited literature on policy changes have therefore not elucidated the mechanisms associating cannabis use and violence since the studies have been conducted in various settings and have used a variety of methodologies (i.e., quasi-experimental difference-in-difference analysis, quasi-experimental, multi-group interrupted time-series design). Globally, supporting studies for both paradigms have assessed how crime is related to the density of cannabis outlets or they have examined state-level changes. Using more rigorous methodologies, some authors have also considered pre-legalization trends in their analyses and controlled for confounding factors, providing better quality evidence for both mechanisms. More thorough investigations are still warranted.

## Discussion

Considering international cannabis policy changes, this focused review aimed to revise the evidence on the association between cannabis use and violence as well as to examine the potential mechanisms involved. Available evidence from meta-analytical studies in youths, intimate partners, and individuals with SMD have shown that there is a global moderate association between cannabis use and violence, which may be stronger in the latter more at-risk population. Though, not only is any type of use of cannabis associated with violence, but preliminary data has highlighted a potential dose-response relationship with larger effects in more frequent users. In this sense, the association between cannabis use and violence is not to be overlooked.

Of interest, positive associations between cannabis use and violence have also emerged in more recent studies following these meta-analyses. For instance, scholars have observed an association between cannabis and violence in intimate partners [e.g. (103–105)]. Our team conducted four additional studies to elucidate the association using more robust methodological strategies and well-known databases in youth populations from the Quebec Health Survey of High School Students (106) and Longitudinal Studies of Child Abuse and Neglect (107) as well as in samples with SMD from the MacArthur Violence Risk Assessment Study (108) and Clinical Antipsychotic Trials of Intervention Effectiveness (CATIE) (109). Beyond associational research, our studies using longitudinal designs were conducted in the aim to further understand the direction of the cannabis-violence association as solely few investigations have been carried out on the matter (27, 31, 33, 107–110). Our studies on psychiatric samples have supported the finding of a unidirectional association between cannabis use and violence (108, 109). In this regard, our research team has recently re-analyzed data from the NIHM-funded CATIE trial. In a sample of 965 patients followed for 12 months, a cross-lag model was implemented to examine the association between cannabis use and violent behavior. Results showed that persistent cannabis use predicted subsequent violent behavior, while the reverse relationship was not significant. Results remained significant after controlling for alcohol and stimulant use. As such, this analysis of longitudinal data showed a unidirectional association between cannabis and violence in schizophrenia (109). On the other hand, our study on adolescents also supported a reverse relationship, that is that externalizing behavior in youths may lead to the subsequent use of cannabis. Hence, using developmental joint trajectory models, it was found that higher levels of trait aggression at ages 10 to 16 were associated with cannabis use at 16–18 years old (107), which supported some scholars’ claim that the association is bidirectional (27, 111). This highlights the importance of better understanding the direction of the association.

Although the mechanism associating cannabis and violence remains to be clearly resolved, a variety of strategies should be implemented in order to reduce the negative impacts of cannabis legalization (82). From a biological perspective, as CBD is more reliably associated to therapeutic properties (such as neuroleptic, relaxant and neuroprotective effects), increasing CBD content may prove to be a sustainable strategy to mitigate cannabis-induced harms (112). Nevertheless, the effects of CBD on violence remain unknown. From a social perspective, preventative measures and intervention programs on mental health and risk behavior should be implemented in school settings since youths remain predominantly susceptible to the detrimental effects of cannabis. They should be provided critical educational information for decision-making and discouraged from initiating and adopting more chronic patterns of use (113). Awareness should be prioritized among professionals (e.g. social workers, educators, clinicians) who are in contact with more vulnerable or violence-prone populations. Professionals should take the necessary measures to further diffuse their knowledge through psychoeducation to their treating individuals. Markedly, efforts should be made to deter violence-prone populations from using cannabis. These at-risk populations include samples from forensic and carceral settings. Noteworthy, in comparison to other drugs, lifetime and regular cannabis use remains the highest drug of use in inmates and the highest drug at time of offence (114). In this sense, crime and substance misuse comprise public health issues for criminal offenders who are released from carceral settings. Interventions should ultimately aim to decrease post-release risky behavior (e.g. cannabis use) among inmates or forensic patients returning to the community (115). Mental health clinicians should screen their patients for cannabis use patterns and related adverse effects of aggression (82). Until a secure exposure pattern (e.g. quantity of cannabis, potency level) is determined by research, withholding from regularly using cannabis may be a better option in these at-risk and vulnerable populations. Moreover, evidence-based treatments and interventions, such as contingency management, relapse prevention, motivational interviewing, and cognitive behavioral therapy showing promising results (116), should be offered to those with problematic cannabis use.

## Limitations

Albeit the important contributions brought forth by the current literature, several limitations must be acknowledged. Upon reviewing the limited available evidence, one important discrepancy involves the heterogeneity among studies. For instance, studies used heterogeneous methods to measure and define violence. Accordingly, it becomes difficult to ascertain whether different constructs of violence were investigated. Further examinations into the essence of the construct should be considered for future research. Of importance, it is necessary to better understand the direction of the cannabis-violence association. In this regard, longitudinal studies should further investigate the direction of the association. Regarding the literature pertaining to policy changes, particularly for recreational cannabis, the vast heterogeneity surrounding study methodologies restrict our ability to precisely evaluate the mechanism associating cannabis and violence. A further predominant limitation in the literature regard the assessment of cannabis exposure/use patterns, such as the type of product consumed (edible, joint, beverages), number of products consumed, dosage, frequency, and THC to CBD ratio, which limits our ability to accurately determine how THC may be associated with violent tendencies. This information in relation to violence will be particularly important to define in the context of public health strategies since legalization aims at the regulation of dosage and potency of the products. This is more so important as health promotion strategies enhance health literacy by providing reliable evidence-based research.

## Conclusion

In all, evidence-based research from meta-analyses have indeed shown that cannabis is associated to violence and therefore measures should be taken to mitigate the risk. Nevertheless, there remains questions as to the direction of the association and the potential mechanisms involved, which may be answered with the changes observed following the liberalization of cannabis. Hence, biopsychosocial research should continue to monitor the association following policy changes more thoroughly by examining different types of violent outcomes. Research should account for trends before legalization and consider the profiles of individuals using cannabis before and after legalization. This methodological consideration has been lacking in most studies in the literature. Moreover, since meta-analytical evidence has found an association between cannabis use and violence in intimate partners, further data on post-liberalization prevalence for dating and intimate partner violence is warranted. Similarly, studies on the effects of cannabis policies in at-risk populations such as individuals with SMD and prisoners leaving carceral settings is necessary. Additional biological studies using neuroimaging, for instance, are currently needed to further shed light into the mechanisms associating cannabis and violence. If causation is established, it will be more so crucial to determine a specific type of exposure pattern (e.g. quantity of cannabis consumed or its potency level) that may be more associated to violent tendencies. For all these reasons and considering the recency of policy changes on cannabis, further methodologically-sound research using longitudinal designs should examine the effects that cannabis may have on different forms of violence and seek to evaluate the trends that emerge in different populations. This should be done while evaluating the effects of possible confounding factors (e.g. other substance use, psychopathic traits).

## Author Contributions

AD, LD, and SP contributed to study planning and design. LD and MA conducted the literature search. LD wrote the manuscript. All authors contributed to the article and approved the submitted version.

## Funding

No specific funding was awarded for this research. LD is holder of a doctoral scholarship from the Fonds de Recherche du Québec en Santé. SP is holder of the Eli Lilly Canada Chair on schizophrenia research. AD is holder of a Junior 2 salary award from the Fonds de Recherche du Québec en Santé.

## Conflict of Interest

The authors declare that the research was conducted in the absence of any commercial or financial relationships that could be construed as a potential conflict of interest.
